# “You never know who are Sami or speak Sami” Clinicians’ experiences with language-appropriate care to Sami-speaking patients in outpatient mental health clinics in Northern Norway

**DOI:** 10.3402/ijch.v75.32588

**Published:** 2016-11-10

**Authors:** Inger Dagsvold, Snefrid Møllersen, Vigdis Stordahl

**Affiliations:** 1 Sami Norwegian National Advisory Board on Mental Health and Substance Abuse (SANKS), Finnmark Hospital Trust, Karasjok, Norway; 2 Centre for Sami Health Research, Department of Community Medicine, Faculty of Health Sciences, UiT – The Arctic University of Norway, Tromsø, Norway

**Keywords:** Sami, mental health, qualitative study, language-appropriate services, language switch, equitable health services, Norway

## Abstract

**Background:**

The Indigenous population in Norway, the Sami, have a statutory right to speak and be spoken to in the Sami language when receiving health services. There is, however, limited knowledge about how clinicians deal with this in clinical practice. This study explores how clinicians deal with language-appropriate care with Sami-speaking patients in specialist mental health services.

**Objectives:**

This study aims to explore how clinicians identify and respond to Sami patients’ language data, as well as how they experience provision of therapy to Sami-speaking patients in outpatient mental health clinics in Sami language administrative districts.

**Method:**

Data were collected using qualitative method, through individual interviews with 20 therapists working in outpatient mental health clinics serving Sami populations in northern Norway. A thematic analysis inspired by systematic text reduction was employed.

**Findings:**

Two themes were identified: (a) identification of Sami patients’ language data and (b) experiences with provision of therapy to Sami-speaking patients.

**Conclusion:**

Findings indicate that clinicians are not aware of patients’ language needs prior to admission and that they deal with identification of language data and offer of language-appropriate care ad hoc when patients arrive. Sami-speaking participants reported always offering language choice and found more profound understanding of patients’ experiences when Sami language was used. Whatever language Sami-speaking patients may choose, they are found to switch between languages during therapy. Most non-Sami-speaking participants reported offering Sami-speaking services, but the patients chose to speak Norwegian. However, a few of the participants maintained language awareness and could identify language needs despite a patient's refusal to speak Sami in therapy. Finally, some non-Sami-speaking participants were satisfied if they understood what the patients were saying. They left it to patients to address language problems, only to discover patients’ complaints in retrospect. Consequently, language-appropriate care depends on individual clinicians’ language assessment and offering of language choice.

The Indigenous population in Norway, the Sami, living in Sami language administrative districts^1^, have since 1990 had a statutory right to receive equitable health care, including an extended right to speak and be spoken to in the Sami language when receiving health services (1). Shared language is a prerequisite for verbal communication, and it is well recognized that when patients speak their preferred language in therapy, it enhances mutual understanding, a good therapeutic relationship between patient and clinician, and may improve the quality of therapy (2–7). Language barriers are common causes of communication problems and clinicians’ failure to understand minority-language patients (6,8–11). Therefore, clinicians’ ability to assess language needs, offer a language choice and evaluate the impact of language in therapy is vital in the provision of language-appropriate care. However, language assessment is complex; language proficiency and level of fluency are not easily defined and may vary whether it is clinician- or patient-assessed. Furthermore, patients’ language use and preferences may vary with relation to interlocutor or topic of conversation (7,12). The purpose of this study is to explore how clinicians deal with language-appropriate care with Sami-speaking patients in specialist mental health services.

Language-appropriate health care for immigrants in southern Norway and for minority-speakers in other countries, for example, Canada, Wales and the USA, is reported to be insufficient. Identification of patients’ language data and offers of language-congruent services or interpretation are poorly implemented (2,5,9,13,14). In a Sami context, there is limited knowledge about clinicians’ experiences with provision of language-appropriate care within specialized mental health services towards Sami patients. Language surveys show considerable variations in the possibilities to use Sami in local health care. Sami majority areas have more Sami-speaking health personnel, but even there service users are dissatisfied with the possibilities to speak Sami when receiving health care (15–17). The lack of Sami-speaking clinicians and professional Sami interpreters is reportedly the main challenge, both in local and specialized health services (15,16,18–21). We have identified two studies of language-appropriate health care for Sami patients. The first, a study of general practitioners (GP) practices, indicates that Sami patients are not offered GP services in Sami (22). The second, a study of specialized mental health services in a psychiatric hospital in Northern Norway, showed that Sami-speaking patients are not always identified as Sami speakers and only occasionally receive therapy in Sami (23). Reports and research to date are limited but indicate that language-appropriate health care for the Sami in Norway is inadequate.

This study aims to explore how clinicians identify and respond to Sami patients’ language data, as well as how they experience provision of therapy to Sami-speaking patients in outpatient mental health clinics in Sami language administrative districts.

## The Sami

The Sami population resides in Norway, Sweden, Finland and the Kola Peninsula in Russia, and is estimated to be about 100,000 people.^2^ The majority, roughly 40,000, live in Norway.^3^ From the mid-19th century^4^, the Sami in Norway experienced a 100–150-year-long period of linguistic and cultural oppression and harsh assimilation policy, leading to among other things language shift among many Sami (2,5,24). The Norwegian Sami policy has gradually shifted from an assimilation ideology, and Sami society is now being revitalized. In Norway, the Sami were formally acknowledged as an Indigenous people in 1990 and they have a constitutional right to maintain and develop their language, culture and way of life (25).

## Sami languages in Norway

The Norwegian Constitution section E, Human Rights, §108^5^ gives Sami and Norwegian languages equal worth and status. There are three main Sami languages: Northern Sami^6^ (26), Lule Sami and Southern Sami, with several dialects within each language (27). The exact number of Sami speakers is unknown; estimates vary between 23,000 (18) and 35,000 (21,28). Furthermore, the exact number is difficult to determine because “Sami-speaking” is not defined in terms of fluency (21,28). Sami language competence varies between generations, family members and locality, since the intensity of assimilation varied in periods and between Sami areas. Most Sami speakers are assumed to be bilingual (29–32). The number of monolingual Sami speakers is assumed to be small, predominantly pre-school children, persons with intellectual or cognitive disabilities and senior citizens (33).

## Language rights in health care for the Sami

Health care for the Sami is integrated in the Norwegian public welfare state system, where they are entitled to receive equitable health services^7^ (34). Several national laws, notably the Patient Right Act and the Sámi Act, confirm Sami patients’ right to speak Sami in health care settings. The Sámi Act stipulates an extended right to use Sami in local, regional or state public bodies (here: health institutions) in the Sami language administrative districts (1,18). The Health Trusts Act emphasizes that specialized health services are responsible for safeguarding Sami patients’ extended right to use Sami in specialist health care (18). However, health institutions are not obliged to employ Sami-speaking clinicians; it is sufficient to offer an interpreter (35,36). According to the Health Personnel Act, clinicians are responsible to fulfil the Patient Rights Act (13,18,21).

## Material and method

### Design

We chose a qualitative design with individual interviews to explore issues of which we have limited knowledge, narrated by clinicians with relevant experience (37).

### Recruitment procedure

The study aimed to include clinicians providing care to Sami patients in outpatient mental health clinics. We requested seven mental health clinics serving patients within Sami language administrative districts^8^ in Northern Norway for permission to recruit participants among their clinicians. Three clinics consented, all located in the northern Sami area. Information meetings and letters containing information about the study, including the interview topics and an invitation to participate, were distributed to 60 therapists in August 2012–November 2013. An inclusion criterion was experience with provision of mental health care to Sami patients. Clinicians interested in participating submitted the consent form directly to the first author, who contacted them for an appointment.

### Sample

A total of 20 clinicians^9^ participated in the study, of which 9 were men and 11 were women. Participants’ age varied from mid-20s to mid-60s. Of the participants, 10 were qualified nurses, social workers, physiotherapists or occupational therapists, and another 10 were psychologists or psychiatrists. Their experiences from mental health care ranged from 2 to about 40 years. Three had some kind of specialized training in cultural studies. Eleven participants identified as Sami and nine as non-Sami. Residency in the Sami area ranged from 1 to approximately 60 years. Five spoke Sami fluently and could provide treatment in Sami, while 15 were unable to provide treatment in Sami because of no (n=10) or limited (n=5) Sami language competence.

### Data collection

The interviews, conducted by the first author, took place at the participants’ chosen location; their workplaces, and lasted from 50 to 140 minutes. The semi-structured interviews were based on a thematic interview guide including topics relevant to the aim of the study: participants’ language awareness with Sami-speaking patients, experiences of provision of language-appropriate mental health care to Sami-speaking patients and the use of Sami in therapy. The questions were open-ended and the order was flexible. The participants were encouraged to talk freely, draw on their own experiences and discuss issues that interested them. All interviews were in Norwegian because the interviewer did not speak Sami fluently. For Sami-speaking participants, the use of Norwegian may have limited free talk. A bilingual interviewer might have accessed other stories about their experiences. The interviews were audiotaped and transcribed verbatim. To safeguard participants’ confidentiality, their name, age, occupation and other personal^10^ details were not included in the transcripts or the presentations of findings.

### Analysis

The transcribed texts were analysed using an inductive approach, according to thematic text analysis inspired by systematic text reduction (37–39), as follows:

- All transcriptions were read several times to obtain an overall impression of “what they were talking about,” followed by reading of the texts in relation to the aim of the article: exploring how clinicians identify and respond to Sami patients’ language data, as well as how clinicians experience provision of therapy in Sami or Norwegian in outpatient mental health clinics in Sami language administrative districts.
- Meaning units were identified representing aspects relevant to the research question. The meaning units for each participant were condensed and coded, which reduced the amount of text without losing the meaning.
- The codes were systematized and categorized across the sample. Related codes were sorted into themes and subthemes.
- Finally, we formulated short texts, summarizing our interpretations of each theme.

The first author read all the interview texts, and selected half of the interviews for the last author to read. The first author formulated code groups and themes, which were introduced to the co-authors, along with selected quotations. The code groups and themes were modified and further developed by all authors in cooperation. The analysis was a process of reading and re-reading, formulation of themes and subthemes and selection of quotations suitable to enlighten the themes and represent the participants’ stories about their experiences.

The findings are presented as experienced by participants who could and those who could not provide therapy in Sami. In both groups, there were differences regarding participants’ characteristics, such as gender, age, education, language, ethnic background and time of residency in Sami- or Norwegian-dominated areas. Characteristics other than Sami language are not mentioned here due to the small number of participants and confidentiality. A different and/or broader demographic sample might have resulted in different findings but require a broader sample and different methods.

### Ethical approval

The research protocol was approved by the Regional Committees for Medical and Health Research Ethics (REC)^11^ and was conducted in accordance with the Helsinki Declaration of 1975, revised in 2008.

## Results

The presentation of findings is based on the text analysis and illustrated by selected quotations. From the analysis, we identified two main themes and five subthemes (see Table I).

**Table I:** Results

I Identification of Sami patients’ language data When is Sami patients’ language data identified. Approaches for Sami language identification.
II Experiences with provision of therapy to Sami-speaking patients Provision of therapy in Sami Offering referral to Sami-speaking services No offer of language choice

### Identification of Sami patients’ language data

The findings in this study showed that the participants identified Sami patients' language data (language proficiency and/or preferred therapy language) either through health institutions' standardized routines, by varied individual approaches, randomly or not at all.

#### When is Sami patients’ language data identified?

Prior to treatment start, referrals from GPs are, according to seven participants (four Sami speakers), the only tool that occasionally informs them about Sami language data, at least when the referring doctor is a “Sami-speaking doctor”. However, most participants (13, including one Sami speaker) found that referrals have no information about Sami patients’ language proficiency or preferred therapy language.

Language-appropriate services, particularly interpreter services, were not organized prior to admission. Sami-speaking patients were most often not assigned to Sami-speaking therapists prior to admission, as stated by one:

Patients don't get the chance to choose a Sami-speaking therapist […] Never seen anything about that in referral letters, I've been involved in admissions and I've never seen anything like that.

Most of the participants were not aware of Sami patients’ language data and had not prepared for a language choice prior to initial contact. Consequently, the participants could decide for themselves whether to identify language or not when they met the patient.

At the outset of therapy, the anamnesis is an institutional tool that can identify language proficiency, according to five participants (one Sami speaker). However, the anamnesis is not obligatory to use. One non-Sami-speaker stated that:

We do ask about the language in the anamnesis […] but with Sami, I don't know if it's always so much emphasized because it's so obvious that they speak Norwegian. If they come in here speaking Norwegian, we presume they speak Norwegian well, like most Sami.

Another non-Sami-speaking participant emphasized that, since the institution serves a multilinguistic population, it should routinely identify all patients’ mother tongue and preference for therapy language:

[…] it's quite incredible that it's possible to take a whole anamnesis without asking about [which language they prefer in the therapy] […] when we live here in the north, and everyone knows there are so many people with Sami as their mother tongue […] and when the patient is in a crisis […] it's quite natural that what we speak then is the mother tongue, it's what lies deepest in a person, isn't it? […]

These participants reported that even if institutional systems for language identification are available, they are not always used. The participants used various individual approaches to identify language data in the beginning of therapy, or discovered it by chance during treatment.

#### Approaches for identification of Sami patients’ language data

All five Sami-speaking participants reported that they always identified Sami language proficiency and preferred therapy language. Among the 15 non-Sami-speaking participants, nine reported that they always identified, six occasionally identified – and two did not identify Sami patients’ language proficiency. As for preferred therapy language, nine reported that they always identified, two occasionally – and four never identified it (two of them had and two had not identified language proficiency). Identified language proficiency was not always followed up by an identification of preferred therapy language.

The Sami-speaking participants identified language proficiency by asking all patients, alternatively identified themselves as a Sami speaker by greeting in Sami or using their Sami name during the first consultation. Sometimes they knew the patient as a Sami speaker from the local community and therefore asked about the preferred therapy language. Some participants simply started to talk in Sami to a patient they believed to be a Sami speaker and left it to the patient to choose language in replying. The patients’ responses when being spoken to in Sami determined the therapy language to be either Sami or Norwegian. However, Sami-speaking patients could change their mind and prefer to switch to another, or between, language(s) during therapy.

The non-Sami-speaking participants identified language proficiency by asking all, or some, patients in the beginning of therapy. Some asked about Sami language proficiency if they observed, what they considered typical Sami characteristics: the patient had a Sami name, “looked like a Sami”, spoke imperfect Norwegian with a “Sami accent” or if a patient lived in a “Sami area” (Karasjok or Kautokeino^12^). One participant admitted that if patients lived in non-typical Sami area, she might forget:

I certainly know which patients speak Sami. There aren't so many […] I usually ask what their mother tongue is, but it does happen that I forget, it's easier to forget it if they come from the coast.

Some non-Sami-speaking participants said that they might discover both language proficiency and preferred therapy language by chance later in the therapy. One said: “I ask about language if I get a hunch,” without being able to define “a hunch”. Another participant related how the use of Sami poetry and music during group therapies may identify hidden or even forgotten Sami language proficiencies among patients:

[…] we always use poetry and music [in group sessions], in Sami too […] and then there are some [patients] who suddenly think of Sami words they didn't know they knew, they remember they heard them, used them, in childhood […] and while we were listening to a Sami song, there were suddenly others in the group who [said they] understood what the song was about, but they didn't say it at first. Two understood the language, but they couldn't speak it, and another could also speak [Sami] […].

This participant emphasized the importance of language awareness when serving the population in Sami areas because “you never know who are Sami or speak Sami.”

Among the non-Sami-speaking participants, there were also reports of no identification of Sami language proficiency. One stated that:

I don't ask about their mother tongue. As long as the patient speaks a kind of Norwegian that I think of as quite normal Norwegian, it's not an issue.

Those participants who did not identify patients’ preferred therapy language reported that they have assessed the patients’ Norwegian proficiency as satisfactory for communication. They spoke Norwegian without asking the patient about preferences and claimed that they have never experienced language problems during therapy. They trust their patients to address language problems:

[…] I tend to say if you don't understand me, please tell me, so I can use other words in a way that you can understand what I'm saying […] it's very important to have them realize that I have some limitations too, I can't speak their language. […] I want to try to help them and if they want help, they have to help me to help them, you could say, if they can.

The findings indicate that, in most cases, the participants identified language data, ad hoc, by using various individual approaches. A few participants assessed patients’ Norwegian proficiency as good and did not ask Sami patients about language data. They trusted the patients to address language problems.

### Experiences with provision of therapy to Sami-speaking patients

Most of the participants offered a language choice for therapy at treatment start or later in therapy. The Sami-speaking participants offer therapy in northern Sami themselves. The non-Sami-speaking participants reported having three options: referring the patient to a Sami-speaking therapist, using an interpreter or providing therapy in Norwegian without offering a language choice.

#### Provision of therapy in Sami

The five Sami-speaking participants offer to provide therapy in northern Sami at treatment start. In their experience, most Sami-speaking patients are bilingual and respond differently to the offer. Many of their Sami-speaking patients prefer to speak Sami, but some also say it does not matter because they speak both languages equally well or prefer to speak Norwegian with therapists out of habit.

When Sami-speaking patients chose to speak Sami, the participants found communication to be more profound and openhearted. One participant stated:

I think it's an advantage to know the language because to speak Sami with those who prefer that opens up in a completely different way than talking in a second language, and I think that when people can think aloud and hear themselves talk, it can be a good help.

However, all the Sami-speaking participants also experienced that many Sami-speaking patients may reject the offer to speak Sami in therapy. One participant reported:

[…] I've wondered sometimes, because some have been talking Norwegian to me, but I know they can speak Sami. But it hasn't happened, then we don't speak Sami even though I know they speak Sami, so it's strange. But I tell them you can just speak Sami, it's no problem to speak Sami if you want to, but often when they've started a conversation in one language, we tend to continue in that language.

Whatever language the patients choose, they usually switch between Sami and Norwegian during therapy. Sami-speaking patients may choose to speak Norwegian in therapy but switch to Sami when communicating what the participants consider sensitive topics such as “[…] emotions, that's very often in their mother tongue, the language of the heart […].”

Language switch may also appear suddenly in the middle of a conversation without an explanation. According to one of the participants, this is how everyday conversation goes on:

In our world, people are bilingual in many ways… some topics we speak about in Sami, and some topics we speak about in Norwegian […], alternating.

All the Sami-speaking participants said that when a patient switches between languages, they switch as well. The participants have not explored when and why language switch occurs during therapy. One participant stated: “I don't know, I haven't thought about it, I just follow, when the patient switches, I switch as well.” According to these participants, the meaning of language in therapy is not discussed among health professionals at their workplaces. They do not have training in language assessment.

Our findings indicate that the Sami-speaking participants in this study always identify the patient's language proficiency and offer to speak Sami in therapy. In their experience, Sami-speaking patients benefit from using Sami language in therapy because they communicate more easily in Sami. However, Sami-speaking patients may choose to speak Norwegian or switch between languages during therapy.

#### Offering referral to Sami-speaking services

Non-Sami-speaking participants reported having two choices when they identified patients as Sami-speakers: referring them to a Sami-speaking therapist or using an interpreter.

*Offering referral to Sami-speaking therapist*. Eight non-Sami-speaking participants reported that they had offered referral to a Sami-speaking therapist, but that the patients hardly ever accepted the offer. In their opinion, Sami-speaking patients may have several reasons to reject such an offer. Some said the offer is given too late, and that Sami patients are polite and may feel uncomfortable about rejecting a therapist's offer to his face. Some assume that patients may be afraid to lose or delay the treatment if they want to speak Sami in therapy. A few reported that a Sami-speaking patient may refuse referral to Sami-speaking clinician when offered by a non-Sami-speaking participant, but accept to speak Sami when offered by a Sami-speaking clinician. However, the contrary may also happen: Instead of transferring to a Sami-speaking therapist, a Sami-speaking patient may prefer to see a non-local therapist, even if it means speaking Norwegian. One participant reported:

I've offered follow-up care with a Sami therapist, but people don't want to be referred there […] The reason is that Sami community is so small and family ties and kinship are really important, they didn't want others to know they had problems and got psychiatric help.

Consequently, most of the non-Sami-speaking participants continued the therapy in Norwegian with Sami-speaking patients.

A few of these non-Sami-speaking participants reported that even if Sami-speaking patients choose to speak Norwegian themselves, language problems and language switch may occur during therapy. Occasionally, when Sami-speaking patients may struggle to express themselves in Norwegian, some participants may encourage patients to “say in Sami” because they believe it helps to think aloud in their mother tongue, even though they themselves would not necessarily understand what the patients are saying. Two participants reported that they have re-examined patients’ language choice because of language problems. One of them stated:

I had a patient who didn't want an interpreter, but I thought this was quite wrong, this was a patient in a crisis who had a lot of difficulty making himself understood […] then I thought, well, it's quite natural that what you speak then is your mother tongue, it's what lies deepest in a person. […] But the patient can say no, I'm going to speak Norwegian, and he can deny or refuse to tell, or God only knows what reason people have, that's a different matter […] But I felt it was far too difficult, I couldn't reach him and I didn't understand, and the patient couldn't explain what he meant either, he couldn't find the Norwegian words that were good enough to give an explanation of how things were inside him.

This participant was dissatisfied with progress in the therapy, evaluated the patient's language choice as insufficient for therapy, and insisted on transferring the patient to a Sami-speaking clinician, which the patient finally accepted.

These participants emphasized the importance of maintaining a continuous language awareness and evaluating whether patients’ language choice works for therapy or not. One participant emphasized that therapists must have a “double attention” and be aware of possible communication failures because of language difficulties during therapy.

Several non-Sami-speaking participants emphasized that they would prefer Sami-speaking patients to receive therapy in Sami. They considered the lack of therapists, especially psychologists and psychiatrists, who speak different Sami languages as limiting the offer of language-appropriate services:

Sami patients should receive services in their own language but they don't, not in this institution anyway. The offer of services in Sami is predominantly available in the Northern Sami area, and it's poor even here.

The findings indicate that when therapists offer language choice, patients’ responses may depend on when the offer is given, by whom and whether they can choose a therapist with whom they can have a strictly professional relationship, which may be difficult in small communities. A few of these participants evaluate language in therapy, and may re-examine patients’ language choices and insist that patients accept referral to Sami-speaking care. However, these non-Sami-speaking participants found limited opportunities to offer language-appropriate services.

*Offering a Sami interpreter*. Six non-Sami-speaking participants had offered an interpreter in therapy with Sami-speaking patients, but their patients always rejected the offer. The reasons are assumed to be that Sami patients wish to avoid an interpreter with whom they have a non-professional relationship, that they may find it difficult to use an interpreter or that they may find it “kind of humiliating to be asked if they need an interpreter, because that sort of implies they don't even know Norwegian properly.”

None of the 15 non-Sami-speaking participants had used a Sami interpreter in their present positions in the northern Sami area. Two participants had earlier used a Sami interpreter but stated that “it's difficult to use interpreter.” Another participant reported that, in her clinic, the use of Sami interpretation was not an issue.

Potential differences in the application of Sami language by clinicians versus interpreters were not mentioned; however, two non-Sami-speaking participants said they preferred referral to Sami-speaking therapist instead of offering interpretation services.

The findings indicate very limited use of Sami interpreters. Only one participant reported that she evaluated and re-examined patients’ rejection of interpretation. The example may indicate that even though a patient has rejected an offer of an interpreter, a language need may appear.

#### No offer of language choice

Seven *non-Sami-speaking participants* (four did not identify preferred therapy language) claimed that they have never found it necessary to offer a choice of therapy language because they never have met a monolingual Sami-speaking patient, and judge Sami-speaking patients to be fluent in Norwegian. These participants have not experienced problems understanding Sami-speaking patients during therapy. One of them stated:

I very rarely have problems [with understanding] […] even one patient, I took over a patient, an elderly person, first [me and my colleague] started together and then I got this patient alone and there haven't been any problems. He accepted it, in the beginning he felt he ought to ask my colleague what I said, but when my colleague wasn't there, he understood everything perfectly. I speak very clearly […] and if I speak slowly, […] it helps.

This participant, who does not routinely identify patients’ language preference, stated that when he can understand the patient, it is unnecessary to offer a language choice for therapy.

These participants reported that Sami patients have not addressed language problems. However, one participant did not discover a language problem until being told that a patient had asked to see another clinician because of language problems. Another participant recalled one incident where a language problem was not identified until a discharge letter from another institution revealed it:

[…] Actually, there was a patient who never said that in our talks, but then I got a discharge letter from another institution, I read that [the patient] thought that [the therapist] talked in such a strange way and was difficult to understand. And that surprised me! We had had lots of long conversations, and it had never been an issue when we talked, things went fine. […] but in fact the patient may not have understood everything I said, but that wasn't the impression I had, as the conversation went very smoothly […].

These findings indicate that some participants are satisfied with the communication if they understand what the patient says, and that they have not identified patient-assessed language problems during therapy. However, patients’ language problems have been discovered when patients have complained to others or in retrospect. Consequently, potential language needs may remain unidentified and language-appropriate services have not been provided.

## Discussion

This study aimed to explore how clinicians provide language-appropriate mental health care for Sami patients by investigating whether and how, clinicians identify and respond to Sami patients’ language proficiencies and preferred therapy language.

The participants in this study reported that even if institutional systems for language identification are available, they are not obligatory and not always used. This leads to insufficient registration of patients’ language data and inadequate organization of language-appropriate services prior to admission for patients in need of specialized mental health care. Consequently, clinicians can decide for themselves whether to identify patients’ language data or not when they meet patients. In most cases, the participants identified Sami language data ad hoc by using various individual approaches. Sami-speaking patients were in most cases offered some kind of Sami-speaking services. However, Sami-speaking patients may choose to speak Norwegian or switch between languages during therapy. Both Sami- and non-Sami-speaking participants experienced language switch during therapy but had not clear ideas of when and why this occurred. Seven participants did not find it necessary to offer Sami-speaking services and trusted the patients to address language problems. Our findings indicate that provision of language-appropriate care to Sami patients depends on whether individual clinicians explore and assess their patients’ language proficiencies and preferences during therapy, hold continuous language awareness and evaluate whether the chosen language works for therapy or not.

Assessment of patients’ language proficiency and language needs is a complex matter. Lack of objective criteria and clear definitions of fluency in mother tongue as well as in the second language complicates the assessment even more. When clinicians assess patients’ language data based on what they perceive as “Sami characteristics”, or local places’ “ethnic rumours” (40), this may indicate ethnic affiliation but entail a risk of maintaining stereotypes about a group of people. Using stereotypical characteristics is an “accidental and unreliable method, based on old-fashioned and static ideas of who are likely to be minority speakers” (6). Furthermore, using personal, local knowledge from social networks, based on knowing “who's who” (6), may well indicate patients’ language proficiency, but does not ensure identification of the preferred therapy language nor the need for language-appropriate care. When clinicians trust stereotypical assumptions, or that they will get “a hunch” about patients’ language proficiency or that patients will request language-appropriate care, language preferences may remain unidentified and patients may not receive a language choice for therapy.

Most Sami-speakers are bilingual (29–32) but bilingualism is neither unambiguously nor easily defined (41). People may describe themselves as bilingual, but “the term does not describe the individual's level of fluency […]” (42). Bilinguals may appear as fluent in the majority language and “it is often assumed that individuals who speak [Norwegian] on an everyday conversation, do not require health interpretation” (7). However, being “fluent in a language varies from individual to individual and a person's fluency in both languages can fluctuate during life, as a result of changes in their circumstances” (42). As for the Sami, the level of fluency in Sami language necessary to be accepted as Sami-speaking has not been defined (21,28). In addition, findings in a previous study (12), as well as in the present study, indicate that Sami-speaking patients switch between languages depending on with whom they talk, and when talking about emotional issues. Bilingualism and the use of language switch in different situations may conceal language needs (42). Therefore, assessment of language needs is emphasized as particularly important when bilingual patients speak some majority language, because language needs may not be obvious (43).

Questions about language proficiency often dichotomize language ability as either - or. Either the patient need an interpreter, or he speaks, or claim to speak, the majority language sufficiently well for therapy. In our study, some participants assessed Sami-speaking patients' Norwegian proficiency rather than their need for Sami-speaking care. This might conceal the need for Sami-speaking services.

Clinicians may lack skills to assess language proficiency (43), and they often overestimate patients’ ability to understand and communicate (7). Some of our participants found that their patients, whom they assessed to speak good Norwegian in therapy, had complained about language problems to other people. This concurs with Sørlie and Nergård's study (23), where the therapists were more satisfied with the communication than were the Sami patients. The Sami patients were skilled in Norwegian, but “their ability to express complex emotions [in Norwegian] may have been more limited than the therapists realized” (23). Patient's Norwegian proficiency may also be more limited than what the patient himself realized.

Patient's assessment of language needs is in line with the principles of patient-centred care (7). However, when health services leave the responsibility to patients, it may reinforce patients’ feeling of shame or being a burden (4,11). Limitations of patient assessment are that patients may overestimate their language skills or may continue to use the majority language instead of admitting limited fluency in the second language (42). It is assumed that language congruence, where clinician and patient share a common language, enhances the quality of interaction (7). Still, as our findings indicate, Sami-speaking patients may assess their Norwegian proficiency as satisfying for therapy and prefer to speak Norwegian with Sami-speaking therapists. However, even though patients have assessed their language skills and chosen the therapy language themselves, clinicians should evaluate the significance of language and maintain continuous awareness to identify language needs and address language problems.

Actually, clinicians are the ones who hold a language need because they depend on high-quality communication to enable them to provide a high-quality mental health care. This agrees with the “active offer” principle, which moves “the responsibility […] from the user to ask for services to the services to provide them” (44). An “active offer” in a Sami context would mean provision of health care in all Sami languages without patients having to ask for it. Mandatory use of standardized routines in the identification of patients’ mother tongue and preferred therapy language might serve as a step to improve language-appropriate services for Sami patients.

The lack of mandatory, routine language identification, leaving the assessment to clinicians, emphasizes the significance of awareness towards the power imbalance that exists within health care provider and patient relationship (45). Services depending solely on the individual clinician's knowledge, attitudes and choice of actions are vulnerable and do not necessarily ensure recognition of language needs and an offer of language-appropriate services (6,8,43). Lack of standardized routines and objective criteria to identify language needs jeopardizes the right to receive equitable health services (43). Lack of language-appropriate services is a violation of the Norwegian Patient Rights Act (13). In a Sami context, when health institutions do not offer language-appropriate services to patients with a right and need to speak Sami, it also violates the Sámi Act. The findings indicate a disparity between Sami patients’ statutory rights and actual health care available in Sami.

## Methodological considerations and limitations

The sample, which was limited to therapists working in the northern Sami area because institutions in other areas refused to participate in the study, represents a potential source of selection bias. Therapists working with, for example, Lule or southern Sami populations might have other experiences due to demographic, linguistic, individual and contextual differences, as well as differences in health services. The study findings are therefore not generalizable or valid for mental health services for the entire Sami population. The study does not comprise information about mental status or possible interconnections between language use and mental health status.

The study was conducted in Norwegian because the interviewer (the first author) did not speak Sami sufficiently well to conduct interviews in Sami. As the results show, five participants were fluent in Sami. A Sami-speaking interviewer might have increased recruitment of Sami-speaking participants and could have explored and discussed the issues in more detail with them. A broader sample and interviews in both Sami and Norwegian might have revealed a broader range of meaning units associated with the importance of the Sami language in mental health care.

We have no interaction data, and we have not interviewed our participants’ patients. Therefore, we do not know how many of them would have preferred to speak Sami; nor do we know whether the participating therapists reflect their patients’ experiences as the patients would have expressed them. We consider our findings to be transferable to health care involving bilingual patients and/or therapists because language is highly significant in communication and mutual understanding.

## Conclusion

Our study indicates that Sami patients’ language proficiency, both in mother tongue and other languages, and preferred therapy language are not systematically identified prior to treatment. Our study demonstrates that clinicians have to deal with identification of language competence and preferred therapy language, as well as organize Sami-language services ad hoc when patients arrive. This complicates the provision of therapy in the patient's preferred language. Our findings correspond with those of Nystad et al. (22) and Sørlie and Nergård (23) and indicate that Norwegian health care needs to improve organizational systems and enhance clinicians’ awareness of Sami patients’ language needs.

We suggest that clinicians maintain continuous language awareness, evaluate language choice and assess language needs during therapy. This may enhance identification of language needs even if a patient has decided against speaking his or her mother tongue in therapy. We use the phrase “continuous language awareness” to emphasize that language is not identified “once and for all” on admittance, but is an ongoing process, including attention to language switch and the significance of language in itself in therapy. We also suggest that language-appropriate services rest on the principle of “active offer”, which emphasizes that health services hold the responsibility.

The findings show that language-appropriate services are a complex matter; their aim is not only to identify language data and offer language choice, but also to accept Sami-speaking patients’ right to reject the offer of Sami as therapy language, while at the same time maintaining language awareness and assessment of language needs.

## Clinical recommendations

To improve language-appropriate care for Sami-speaking patients, health services need to systematize the identification of language data and organization of language-appropriate services in line with the patient's preference. Mandatory identification and documentation of language needs among minority-language patients, preferably before the initial consultation, is recommended to ensure access to high-quality, equitable health care. Provision of language-appropriate services depends on the recruitment and presence of minority-speaking clinicians and organization of interpreter services. Sami patients may avoid admitting limited Norwegian proficiency and choose to speak the majority language for a number of reasons. Health services must ensure that organizational factors, for example, lack of minority-language therapists and inadequate routines, do not prevent patients from agreeing or demanding to speak their mother tongue. Clinicians should also be aware of the significance of patients’ first language even if they choose or agree to speak the majority language in therapy. As one participant said: you never know who speak Sami.

## Further research

Assessment of language proficiency and language needs is a complex matter and requires competence in language use and assessment. Further research may benefit from a multidisciplinary approach. This study had a small number of Sami-speaking participants and should be followed up with a broader demographic sample to reveal experiences with the Sami language in mental health care, also in other Sami areas in Norway and in other countries. Sami-speaking and bilingual researchers should conduct further research to explore Sami-speaking and bilingual therapists’ experiences in their first language, which may provide more detailed descriptions and discussions. The significance of Sami languages in relation to mental health care for Sami outside the core Sami areas, such as Lule or South Sami populations, will probably add important understanding to the topic.

This study concurs with findings in a previous study showing how Sami-speaking patients switch between Sami and Norwegian in therapy (12). This raises questions about the significance of language switch in therapy. When and why does it occur? Does language switch influence the therapy process, and even the outcome? We suggest further research to explore the significance of language and language switch in therapy.

Furthermore, the use of Sami music or poetry in therapy may demonstrate acceptance of Sami identity and allow the patients to admit, or remember, Sami language competence. Sami patients may have forgotten, or chosen to hide, their Sami language proficiency, but when processing childhood memories, the Sami language may be important to them. This calls for further investigation of the significance of mother tongue for minority speakers, when processing traumatic events in the majority language.

Language awareness and language-appropriate care enhance health services and outcome for minority-language patients (2,4,6). For the Sami population, knowledge about the significance of therapy language for health status is limited. One example in our study is that Sami-speaking participants found that patients speaking Sami allowed for more nuanced descriptions and more profound understanding, which probably improved therapeutic communication. We suggest further research to establish more knowledge about this important issue.

## Footnotes

1 Sami language administrative districts include the municipalities Tana, Nesseby, Porsanger, Karasjok and Kautokeino in Finnmark; Kåfjord and Lavangen in Troms; Tysfjord in Nordland; and Snåsa and Røyrvik in Nord-Trøndelag.

2 This study is limited to Sami in Norway.

3 There are no census data on the number of Sami inhabitants because it is prohibited to register individual ethnicity data in Norway. The estimate of 40,000 has remained unchanged since 1970.

4 The process of Christianization and assimilation started even earlier, in the 17th and 18th centuries, in the south Sami areas.

5 Former § 110

6 Northern Sami is the language spoken by most Sami in Norway.

7 Equitable services include equal access to treatment and equal treatment outcomes in health care, irrespective of place of residence, economy, social status, age, sex or ethnic affiliation.

8 Where the Sámi Act gives Sami patients an extended right to use Sami.

9 We refer to them as participants.

10 All clinicians are named as she or her, and all patients as he or his.

11 REK-number delivers on demand.

12 Two municipalities in the inland in the county of Finnmark with a majority of Sami, often called the “core Sami area.”
